# Psychometric Properties of the Korean Version of the Environmental Health Literacy Scale

**DOI:** 10.3390/ijerph19074079

**Published:** 2022-03-29

**Authors:** Jung-Min Kwak, Ju-Hee Kim

**Affiliations:** College of Nursing Science, Kyung Hee University, Seoul 02447, Korea; jungminkwak@khu.ac.kr

**Keywords:** environmental health literacy, scale, reliability, validity, factor analysis, Korea

## Abstract

The environmental health literacy (EHL) scale evaluates media-specific and general EHL levels in three domains: knowledge, attitude, and behavior. This study aimed to adapt the EHL scale developed by Lichtveld et al. into the Korean language (K-EHL scale) and to verify its reliability and validity. Survey data was collected from 492 adults (19–65 years) residing in Korea. The study process included translation procedures, content validity verification, pre-testing, the actual survey, and statistical analysis for validation and selection of the final items. The scale-level content validity index was 0.92, and one item was removed. Multiple exploratory factor analyses condensed the K-EHL into 2 factors and 38 items. The “Environmental health knowledge and attitude” factor (14 items) measures information, feelings, and thoughts about environmental health. The “Environmental health behavior” factor (24 items) comprises behaviors responding to environmental health. A construct validity (criterion and discriminant validity) was verified using confirmatory factor analysis for goodness of fit (CFI = 0.901, TLI = 0.863, GFI = 0.923, NFI = 0.862, and RMSEA = 0.08). Internal consistency reliability test results showed a Cronbach’s α of 0.81 for the total items. This study is the first to introduce the EHL in Korea, and it also presents a validated evaluation tool. The K-EHL is expected to elucidate EHL levels in Korea. In the future, the EHL scale can be enhanced using this tool.

## 1. Introduction

As global environmental change is rapidly emerging in society, various environmental pollutants are attracting attention as major determinants of health in Korea. Environmental health hazards caused by environmental exposure are often fatal to the human respiratory and reproductive systems. Furthermore, noncommunicable diseases, including ischemic heart disease, cancer, and stroke are frequently caused due to environmental exposures to contaminated water, endocrine-disrupting chemicals, air pollution, and toxic materials [1,2,3]. The World Health Organization estimates that approximately 23% of premature deaths are closely related to environmental factors, and air pollution kills almost seven million victims every year [3]. Environmental exposure can affect humans through various environmental media and pathways, such as food, water, particulate matter (PM), and the living environment [4]. As various environmental health risks occur in daily life, public interest in the environment and healthcare is emerging. In Korea, environmental health issues related to air, food, and water in daily life continue to appear, as can be seen in the headlines: “A humidifier disinfectant scandal”, “Child death accident with hamburger disease”, and “Arsenic contamination of bottled water”. In this regard, it is important to evaluate the public’s knowledge of environmental health, as well as attitudes and practices. Health literacy is the most academically important concept in meeting healthcare needs. Environmental health literacy (EHL) is a concept that considers the relationship between environmental exposure and health promotion. Davis et al. [5] defined environmental health literacy as the combination of environmental literacy and health literacy. Research on EHL is of great significance because it has an important effect on reducing exposure to environmental hazards and can ultimately contribute to the promotion of human health from the point of view of individuals and communities [6]. Although the evaluation of EHL is an essential concept in modern society, existing, validated tools are limited in Korea.

While several EHL scales exist, few have been generalized and validated [7,8,9,10]. A previous study by Irvin et al. developed the water environmental literacy level scale (WELLS) and verified its reliability in the USA [7]. However, it only measures the EHL related to a single environmental exposure and water pollutants, so it is difficult to popularize it as a general EHL scale. Perceived pesticide risk and perceived pesticide control scales that measure the literacy of pesticide exposure also have the same limitation as WELLS, and 90% of the participants gave the same response during the validation process; moreover, the scales also showed low internal consistency [8]. In the case of the Environmental Health Awareness Instrument, Ratnapradipa et al. [9] created a general EHL assessment tool that included items related to 11 key areas of environmental health, including air, water, food, and toxic substances. However, there are a few limitations associated with this EHL tool: the total number of items is large (443 items), and hence entire items were only validated through focus group discussions by sections, but verification with community members was omitted. Moreover, the survey results are difficult to generalize because most of the focus group discussions were conducted in one geographic area, which might have a specific environmental issue. 

Lichtveld et al. [10] developed the Validated Scales of General Environmental Health and Environmental Media-Specific Knowledge, Attitudes, and Behaviors (EHL scale) in which items measure EHL centered on three media (i.e., air, food, and water) and general environmental health (EH). Each scale is conceptualized into three domains: knowledge, attitude, and behavior. These three media have daily contact with individuals and are associated with the concerns of the community. Moreover, the validity of each scale was verified by community members of more than two different geographic areas [10]. Despite the presence of a reliable tool, no one has translated it into Korean or verified the psychometric properties of the tools to date. Unlike some other countries, the EHL concept has not yet been generalized in Korea, and there are few validated tools related to environmental health concerns. Therefore, it is necessary to verify the validity and reliability of the Korean version of the EHL scale, considering the various interpretations of EHL among different cultures and groups. The present study aims to increase public interest in environmental health by conducting research using the validated K-EHL tool, which can improve EHL among people in Korea.

The purpose of this study was to adapt the original EHL scale by Lichtveld et al. to the Korean language and culture (the Korean version of the Environmental Health Literacy Scale, K-EHL), and to verify the reliability and validity of the scale among Korean adults [10].

## 2. Materials and Methods

### 2.1. Study Design

This study used a methodological research design to verify the reliability and validity of the Korean version of the Environmental Health Literacy Scale translated into Korean, so that it can be applied in Korea.

### 2.2. Study Population

The targeted study population for verifying the validation and reliability of the K-EHL scale was adults, aged 19–65 years, residing in Korea. The participants were able to communicate in Korean and answer the questionnaire. Those diagnosed with environmental diseases within the last three years were excluded. The minimum sample size required for a factor analysis (FA) is ten times the number of variables [11], and according to another criterion for validation studies, a sample of 300 is classified as good and 500 is very good [12]. Therefore, the sample size was set to 460 in this study, and a total of 500 adults was considered adequate, considering a potential dropout rate of approximately 10%.

### 2.3. Study Instruments

#### 2.3.1. EHL Scale

The EHL scale was originally developed by Lichtveld et al. [10] to measure overall knowledge, attitude, and behavior related to media-specific and general environmental health. This tool involves four scales: air, food, water, and general environmental health. A total of 42 items are rated on a five-point Likert scale, with higher scores representing higher levels of EHL. At the time of development, the scales showed internal consistency, with Cronbach’s alpha from 0.63–0.70.

#### 2.3.2. Korean Version of the Environmental Health Engagement Profile (K-EHEP)

The Korean version of the environmental health engagement profile (K-EHEP), which contains five subscales defined by statements describing pollution and environmental health, was used to evaluate the criterion validity of the K-EHL scale. Originally, the environmental health engagement profile (EHEP) was developed by Dixon et al. [2] to assess how people deal with environmental pollution and environmental health issues. This scale was validated in Korea by Kim [1] through confirmatory factor analysis. In a previous stage of the research, the EHEP was translated and adapted into Korean, and its content validity was established.

### 2.4. Study Procedure

The research process included translation procedures, content validity verification, pre-testing, the actual survey, and statistical analysis for validation and selection of the final items. The study was carried out from March 2021 to February 2022, and the actual survey was conducted from 3 September 2020 to 4 October 2020. We received approval via e-mail from the authors of the original tool to use the EHL and EHEP.

#### 2.4.1. Translation and Adaptation

In the forward translation stage, a professional translator fluent in Korean and English translated the original scale into Korean, considering the definition of terms and cultural relevance. Next, a professor at the Department of Nursing reviewed and revised the draft. After the first revision, the translation was back-translated into English by an environmental engineering expert without seeing the original English version. Subsequently, one native English speaker evaluated the similarity between the back-translated text and the original text. Finally, a professor specializing in environmental health and tool development, two nursing doctoral students, and two nursing master’s students with more than four years of clinical experience reviewed the sentence expression, word selection, and translation consistency, considering the concept of measurement of the K-EHL scale.

#### 2.4.2. Content Validity

The K-EHL scale was presented to a total of nine clinical experts and academic experts (two professors of nursing, one expert in public health, one expert in environmental engineering, four nurses with more than five years of clinical experience, and one expert in health science) to determine whether items appropriately described the property to be measured and the validity of the content. Content validity was measured using the item-level content validity index (I-CVI) and scale-level content validity index (S-CVI) on a 4-point scale with the following responses: 1 = not relevant; 2 = revision required: unable to evaluate relevancy or appears not to be relevant without revision; 3 = relevant but requires modest revision; and 4 = highly relevant and concise [13,14]. If there were six experts or more in agreement, an I-CVI over 0.78 was considered valid, and in the case of an S-CVI, 0.90 was the optimal cutoff value to verify the content validity [15,16].

#### 2.4.3. Preliminary Pilot Study

In this study, a preliminary survey was conducted with 20 adults who met the subject criteria; these respondents did not overlap with the main survey respondents. In the preliminary survey, the average response time of the entire questionnaire did not exceed 30 min. Although it was suitable for understanding the questionnaire items, there was an opinion regarding the structure of the questionnaire. They responded that it was more appropriate to group the items together by measurement domains, such as knowledge, attitude, and behavior, rather than to compose the tool with items for each medium, such as air or water scales. After discussions with experts, the final version was completed using the new questionnaire.

#### 2.4.4. Data Collection and Ethical Consideration

This study was approved by the Institutional Review Board of Kyung Hee University (KHSIRB-21-363). Owing to the COVID-19 pandemic, this study was administered online to adults in the Entrust survey (kr.entrustsurvey.com, accessed on 3 September 2020), which is a web-based platform for recruiting participants who are offered a small reward. The sample size of the study participants was set to 500. Based on this, a total of 500 copies of data were obtained, but 492 valid questionnaires were finally included in the analysis, with eight copies being excluded because responses were insincere or incomplete. To ensure anonymity, the collected data were statistically analyzed by assigning only numerical identification codes to each respondent. Participants were adequately informed about the objectives and the process of the study and had the freedom to withdraw from the research at any time without any prejudice.

#### 2.4.5. Validity and Reliability Testing

Exploratory factor analysis (EFA) and confirmatory factor analysis (CFA) were conducted to evaluate construct validity. Content and discriminant validity were assessed, and the model fit value was evaluated by comparing it with the original tool. To investigate criterion validity, the K-EHEP was measured. Internal consistency was analyzed using Cronbach’s alpha to verify the reliability of the K-EHL scale [17].

### 2.5. Data Analysis

The collected data were analyzed using IBM SPSS AMOS 26.0 for Windows, version 25.0. The general characteristics of the participants were analyzed using descriptive statistics. The items were analyzed using the mean and standard deviations of each item, inter-item correlations, skewness, and kurtosis of the data. To minimize redundancy in the subscales, highly correlated items (>0.80) were discarded [18]. Kolmogorov–Smirnov and Shapiro–Wilk normality tests were performed to evaluate the normal distribution of the collected data [19].

In the EFA process, the scale factors, items in each factor, item factor loadings, and explanatory power were examined. The Kaiser–Meyer–Olkin (KMO) test and Bartlett test of sphericity were performed to determine the suitability of the collected data for factor analysis [20,21]. Principal component analysis was used for factor extraction, and the varimax rotation method was used for factor rotation. The criteria for determining the number of items and factors were based on a scree plot and previous studies, including the original tool. As for factor loading, the item included in the factor was determined when the loading was at least 0.30 [22].

Once the number of factors was determined, the goodness of fit of the model was determined using indices such as χ^2^, degrees of freedom, goodness of fit index (GFI), comparative fit index (CFI), Tucker–Lewis index (TLI), and root mean squared error of approximation (RMSEA) through CFA [23]. Both the EFA and CFA were performed using data from 492 participants.

The discriminant validity of the items was tested by analyzing Pearson’s correlation between the subfactors of the K-EHL scale, which reflects whether the factors are measuring the same concepts [19]. To verify the criterion validity, Pearson’s correlation analysis between the Korean version of the Environmental Health Engagement Profile (K-EHEP) and the K-EHL scale was carried out to check whether there was a significant correlation [24]. Regarding the reliability of the scale, homogeneity was evaluated based on internal consistency using Cronbach’s alpha [25,26].

## 3. Results

### 3.1. Participants’ General Characteristics

In this study, 492 valid data from men and women between the ages of 19 and 65 years were used for statistical analysis. The ratio of males to females was approximately 5:5, the average age of the respondents was 38.97 years (SD = 10.78 y), and 58.7% of the respondents were in their 30s and 40s. Regarding education level, attending or graduating from university accounted for the majority (*n* = 361, 73.4%), and 333 people (67.7%) answered that they were living in a metropolitan area when asked about their current residence. Regarding marital status, 52% of the respondents were married, and those who were not married (48%) were evenly distributed (Table 1).

### 3.2. Item Analysis

The mean and standard deviation of each item were measured, and normality was evaluated using skewness and kurtosis. The average score for each question was 1.79 to 4.59, and the standard deviation was 0.647 to 1.208. The average score of “I have had my indoor air tested”, an air scale item, was the lowest with 1.79 points, and the item with the highest average score was “Secondhand smoking is harmful to health”. Regarding the normality of the data, the absolute value of skewness was 0.04 (minimum) and 1.95 (maximum). In the case of kurtosis, the minimum absolute value was 0.06, and the maximum absolute value was 1.95, thus ensuring data normality [18]. In addition, Kolmogorov–Smirnov and Shapiro–Wilk tests were conducted to evaluate the normality test, and the significance level was higher than 0.05, which satisfied normality (*p* = 0.181). When inter-item correlations for a total of 42 items were tested by dividing them into the 4 original scales (i.e., air, food, water, and general EH scales), the correlation coefficient between items was distributed between 0.01 and 0.58. All correlation coefficients were below the reference value of 0.80, ensuring that none of the 42 items in the K-EHL scale were likely to overlap.

### 3.3. Validity Testing

#### 3.3.1. Content Validity

Of the total 42 items, 37 items of the I-CVI satisfied the standard value of 0.78 or higher, but items 15, 19, 30, 33, and 36 were evaluated as being below the standard. Among them, item 36 (“Cutting a tomato on a cutting board after cutting raw meat without washing the board might lead to cross-contamination and spreading of disease”) had the lowest I-CVI of 0.5 and was removed after considering the opinions of the experts. The S-CVI score was 0.92.

#### 3.3.2. Construct Validity

(1) Exploratory Factor Analysis

An exploratory factor analysis was performed on 41 items, with the exception of one item that was deleted in the process of content validity verification. To verify whether the data were suitable for factor analysis, the KMO and Bartlett test of sphericity were performed. The KMO value was 0.84, and the χ^2^ value of the Bartlett sphericity test was 5846.71 (*p* < 0.001), which is suitable for the analysis [20].

Three factors were extracted from the scree plot of the first round of factor analysis, and the secondary factor analysis was continued by fixing the number of factors to three. As a result, three items (W3_k, W9_b, and W13_b) had factor loadings lower than 0.30, indicating that the items were not statistically significant. In particular, item W3_k (“The 30 government oversees the quality of drinking water in cities around the country”) is considered culturally unadapted because the subject of water quality management differs from country to country. For the same reason, items W9_b and W13_b were discarded. In the third round of factor analysis, after removing three items, the factor loading for each factor ranged from 0.32 to 0.64, and none of the items scored at less than 0.30 (Table 2). The percentage of explained variance for each factor was 15.83% for Factor 1, 11.36% for Factor 2, and 5.9% for Factor 3, with a total explained variance of 33.1% (Table 2).

Factor 1 consisted of 18 items; 8 items measured knowledge (_k) and ten items measured attitude (_a) out of the 3 domains of the K-EHL scale items (i.e., knowledge, attitude, and behavior). In this factor, the items measuring information or facts about environmental health and those asking about feelings or thoughts about environmental health were included.

In Factor 2, 14 out of 16 items measured behavior (_b) and were items related to the behaviors of individuals and communities responding to environmental health. However, two items, G4_a (“I worry about the chemicals I am exposed to on a daily basis”) and G5_a (“I worry about chemicals because they are always bad for my health”), measured attitudes toward the environment. Regarding factor loading, Factor 1 showed a load of 0.406, and Factor 2 showed a load of 0.488 for item G4_a. In the case of item G5_a, the factor loadings were 0.216 for Factor 1 and 0.442 for Factor 2. Even though G5_a has a stronger correlation with the items of Factor 2, both values indicate that they do not have a clearly strong load on either factor [11]. Furthermore, both items describe attitudes about the environmental health using the term “worry” as an expression for measuring cognitive and psychological properties like other items within Factor 1. If the factor loading value is applied as an absolute standard to interpret the analysis result, essential items or the original content of the instrument might be lost. Hence, researchers should consider each item’s features and properties in the process of factor analysis [22,27,28]. As a result, it was judged appropriate to classify two items into Factor 1, considering the relevance and properties of the items.

In Factor 3, all included items measured environmental knowledge and attitudes related to air. Even though these items had a factor loading ≥0.30, the explanatory power of Factor 3 was only 5.90%. This indicated that Factor 3 was not significantly explained. Rather, these items better represented the properties of Factor 1. Thus, we moved the four items to Factor 1 after discussion with the researchers. The final version of the K-EHL scale consists of 38 items and 2 factors. Factor 1 was named “Environmental Health Knowledge and Attitude”, and Factor 2 was named “Environmental Health Behaviors”.

(2) Confirmatory Factor Analysis

Model fit was confirmed by confirmatory factor analysis of the two factors determined from EFA and the items in each factor. The original structure of the EHL scale is a three-factor structure. Table 3 compares the model fit of the factor structure of the K-EHL scale derived in this study and the original three-factor structure.

**Figure 1:**
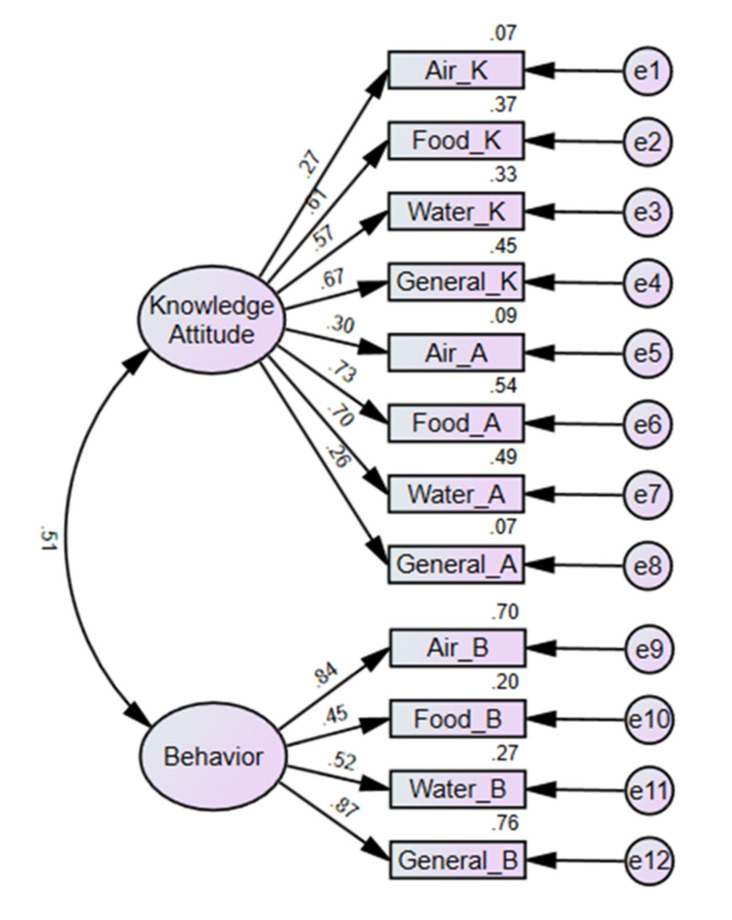
Confirmatory factor analysis of the Korean version of the Environmental Health Literacy (K-EHL) Scale.

As a result, the RMSEA, CFI, and GFI values were aligned with the recommended values [29]. Although the TLI and NFI values were slightly below the standard of 0.90, the other indices of the K-EHL scale showed a better fit than the three-factor model. Finally, the two-factor structure of the K-EHL scale was determined to be the final model, referring to previous studies, where the acceptance criterion of the fitness criterion is not absolute and the judgment of the researcher is important [30]. Figure 1 shows the structural model derived from the confirmatory factor analysis in this study.

(3) Discriminant Validity

To evaluate the discriminant validity of the K-EHL scale, a correlation analysis was performed between the two factors. The correlation coefficient between Factor 1 (environmental health knowledge and attitude) and Factor 2 (environmental health behavior) was 0.29 (*p* < 0.01) (Table 4). This value was lower than the minimum criterion of 0.30, and the concepts measured by the two factors had no significant correlation with each other [31]. In other words, the subscales were interpreted as being separate from one another so as to verify the discriminant validity of this tool.

#### 3.3.3. Criterion Validity

The normality of the response data from K-EHEP was satisfied before performing the analysis. Table 5 shows the correlation coefficients between the K-EHEP and K-EHL total and subscale scores. All the correlations were positive. Regarding the subscales, if the concepts measured in each tool were similar, there was a significant correlation between the two tools. In conclusion, the K-EHL scale measures the concepts of EHL similar to that of the K-EHEP, and criterion validity was established [28].

### 3.4. Reliability

The internal consistency of 38 items in the K-EHL scale was examined; Cronbach’s alpha for the total items was 0.83. By subscale, the Cronbach’s alpha was 0.80 for the Environmental Health Knowledge and Attitude subscale and 0.78 for the Environmental Health Behavior subscale.

## 4. Discussion

This study was conducted to prevent environmental diseases and to promote the health of subjects in the local community. The main properties of EHL were defined as people’s knowledge, attitudes, and behaviors regarding environmental health [6,10,32,33], and the validity and reliability of the EHL evaluation tool developed by Lichtveld et al. [10] was verified for suitability for Korean subjects. The K-EHL scale is a well-prepared tool that consists of 38 items and two factors: “Environmental Health Knowledge and Attitude” and “Environmental Health Behavior”.

In the process of item analysis, the average score of the air scale was the lowest at 2.85 among the four translated scales (i.e., air, food, water, and general EH), and the average score of the air scale in the original study was 2.78, similar to the results of this study. This is also similar to Park et al. [34], who found that women answered that indoor air pollution was the least serious of the 13 environmental problems in a study on environmental health awareness among women. In this previous study, women reported that outdoor air pollution was the fourth most serious problem, but they were unaware of the seriousness of indoor air pollution, such as at home or in the office [34]. Considering the fact that, in the current study, the item, “I have had my indoor air tested,” scored the lowest, it can be seen that the public’s understanding of indoor air environmental health information is at a very serious level. Indoor air pollution is reported to be the main cause of cardiovascular and respiratory diseases, and there is an urgent need to develop an intervention program to improve indoor air quality for vulnerable groups because it causes fatal damage to the cardiovascular function of pregnant women, children, and the elderly [35].

The final version of the K-EHL scale has a different structure from that of the original tool, which is composed of three domains for each medium. Although the results do not exactly match the initial configuration of the tool and the knowledge–attitude–behavior theory, which was the theoretical model in this study, this is consistent with previous studies that have developed a dichotomized questionnaire including cognitive–psychological and behavioral attributes [36,37]. Kim [37] developed the environmental health behavior of female adolescents (EHB-FA) and environmental health behavior tool for female adolescents (EHB-FA). Erzengin and Teke [36] developed environmental attitudes and behavior tools for college students. In Korea, there have been many attempts to analyze the correlation between environmental behavior and environmental awareness, or to identify factors that influence environmental behavior [38,39,40]. Considering these research trends, it is meaningful that environmental health behavior was derived as an independent factor and that environmental attitude and environmental knowledge were combined as one factor in the current study.

In the current study, the criterion validity of the K-EHL scale was verified satisfactorily. Looking at the correlation by subfactor, the more sensitive the response to environmental pollution, the higher the level of environmental health knowledge and attitude (r = 0.58, *p* < 0.01). The higher the overall environmental health engagement, the higher the behavioral level of environmental health literacy (r = 0.80, *p* < 0.01). The relationship between the cognitive, psychological, and behavioral attributes of environmental health is well established. This finding is consistent with Ahn et al. [38], which verified the relationship between environmental knowledge, environmental awareness, and environmental behavior. The provision of environmental knowledge increases environmental interest and environmental importance, which in turn had a positive effect on environmental health practices [37,38,39,40].

However, the current study has several limitations. First, regional characteristics had a narrow influence on the study results because more than half of the study participants were living in large cities (*n* = 333, 67.7%). Compared to large cities where residents mainly live in apartments and are exposed to many pollutants, rural areas are expected to show environmental and cultural differences not only in the air environment, but also in the water and food environment. Second, since the samples were only collected online, there is a possibility that subjects were exposed to more health information because they had relatively little difficulty in using the Internet, which affected the overall EHL score. Third, the result of exploratory factor analysis revealed that the total explained variance is 33.1%, indicating that the three factors are not enough to explain the whole scale [41]. This shows that there exist more diverse factors that contribute to EHL in addition to knowledge, attitude, and behavior. In particular, the explanatory power of Factor 3 was 5.9%, which was far below the standard [41]. Hence, the items constituting Factor 3 were incorporated into other factors based on the judgment of the researcher. However, follow-up studies should be supplemented to improve the explanatory power of each factor and the total explained variance through CFA that includes more diverse factors in other ways. Fourth, in a recent study, when both EFA and CFA were performed, the participants were divided into two groups in advance, followed by EFA with group 1 data and CFA with group 2 data [10,39,40,42]. However, since the same sample was used in both factor analysis processes of the current study, the weakness of the EFA results could have affected the CFA model fit values and standardized factor loading results. Therefore, an additional verification process using a separate sample group is required in future studies to determine whether the K-EHL scale model can be generalized to the general population.

This study is meaningful in that it defines the concept of environmental health literacy, which had not been actively introduced in Korea, and presents a validated evaluation tool. If the public pays close attention to environmental health and environmental health promotion activities, the general level of EHL is expected to improve. Major environmental health hazards are constantly changing, and recently, environmental health issues caused by radon, microplastics, and particulate matter (PM) have become important in Korea. Therefore, in future studies, it is necessary to regularly update the items to measure EHL related to new environmental media based on the K-EHL scale.

## 5. Conclusions

This study translated the Lichtveld et al. [10] EHL scale into the Korean context and evaluated its validity and reliability. The original tools consisted of 3 factors in each media-specific subscale (i.e., air, food, water, and general EH scales) and 42 items. As a result of the multiple factor analysis, the K-EHL scale was condensed into 2 factors and 38 items, and its reliability was satisfied. The “Environmental health knowledge and attitude” factor has 14 items measuring knowledge or thoughts about environmental health. The “Environmental health behavior” factor has 24 items composed of the behaviors of individuals and communities responding to environmental health.

## Figures and Tables

**Table 1:** General characteristics of respondents in the Korean version of the Environmental Health Engagement Profile scale (*n* = 492).

Characteristics	Categories	*n* (%)	Mean (SD)
Gender	Male	243 (49.4)	
	Female	249 (50.6)	
Age (y)	<30	113 (23.0)	38.97 (±10.78)
	30–49	289 (58.7)	
	50–65	90 (18.3)	
Highest level of education	High school or less	70 (14.2)	
	University/college or above	361 (73.4)	
	Graduate school or above	61 (12.4)	
Monthly household income (unit: 1000 won)	<1000	35 (7.1)	
	1000–3000	101 (20.5)	
	3000–5000	159 (32.3)	
	≥5000	197 (40.1)	
Marriage status	Married	256 (52.0)	
	Never married/Divorced or separated	236 (48.0)	
Working status	Employed	353 (71.7)	
	Unemployed	139 (28.3)	
Residence	Metropolitan	333 (67.7)	
	Nonmetropolitan	159 (32.3)	

**Table 2:** Exploratory Factor Analysis of the Korean version of the Environmental Health Literacy (K-EHL) Scale (*n* = 492).

Items (*n* = 38)	Factor 1	Factor 2	Factor 3	Variance (%)
F2_k	0.640	0.169		
F7_a	0.622		0.122	
F1_k	0.613			
W5_a	0.606	0.308		
F5_a	0.599	0.168		
F3_a	0.589	0.136	−0.132	
G2_k	0.570			
W2_k	0.557		0.215	
G1_k	0.510	0.162	−0.245	15.83
W7_a	0.510	0.205	0.210	
F6_a	0.509	0.235		
A5_a	0.482		0.375	
W4_k	0.476	−0.145	0.120	
A1_k	0.475	0.116		
G6_a	−0.437	0.156		
W6_a	0.434	0.279	−0.121	
F4_a	0.419	0.345	0.233	
W1_k	0.317			
A9_b	−0.123	0.596		
A8_b	0.236	0.564		
G8_b	−0.212	0.557	−0.163	
F8_b		0.539	−0.135	
A10_b	−0.205	0.503		
G9_b	0.120	0.497		
W11_b	0.383	0.491	0.214	
G4_a	0.406	0.488		
F9_b	0.233	0.484		
A7_b	0.143	0.482		11.36
W10_b	0.378	0.468		
G7_b	−0.280	0.443	−0.382	
G5_a	0.216	0.442	0.135	
W14_b	0.135	0.429		
W12_b	0.265	0.348	0.149	
W8_b	−0.245	0.330	−0.101	
A4_a	−0.103		0.793	
A6_a			−0.764	
A2_k			0.381	5.90
A3_k	−0.113	−0.133	0.377	
Eigen value	6.02	4.32	2.24	Cumulative (%) = 33.1

Note. A = air scale, F = food scale, W = water scale, G = general scale, K = knowledge, a = attitude, b = behavior; Significant factor loadings are in the shaded backcolor.

**Table 3:** Indices of fit for the original model and the Korean version of the Environmental Health Literacy (K-EHL) Scale model.

	χ^2^ (*p*)	df	CFI	TLI	GFI	NFI	RMSEA
Original	234.385 (*p* < 0.001)	51	0.857	0.814	0.921	0.826	0.08
K-EHL	228.015 (*p* < 0.001)	53	0.901	0.863	0.923	0.862	0.08
Criteria			>0.90	>0.90	>0.90	>0.90	0.05 (good) 0.08 (mediocre) 0.1 (poor)

Note. χ^2^ = Chi-square, df = degrees of freedom, CFI = comparative fit index, TLI = Tucker–Lewis coefficient index, GFI = goodness of fit index, NFI = normed fit index, RMSEA = root mean square error of approximation.

**Table 4:** Correlation matrix by factor of the Korean version of the Environmental Health Literacy (K-EHL) Scale.

	Environmental Health Knowledge and Attitude	Environmental Health Behavior
Environmental Health Knowledge and Attitude	1	0.29 **
Environmental Health Behavior		1

Note. K-EHL, Korean version of the Environmental Health Literacy Scale; ** *p* < 0.01.

**Table 5:** Correlation between the K-EHEP and K-EHL scale by subscales (*n* = 492).

K-EHEP							
		PS	PCI	PA	CEA	PEA	Total
K - E H L	Environmental Health Knowledge and Attitude	0.58 **	0.45 **	0.41 **	0.15 **	0.32 **	0.62 **
	Environmental Health Behavior	0.19 **	0.33 **	0.01	0.65 **	0.55 **	0.80 **
	Total	0.48 **	0.58 **	0.27 **	0.35 **	0.53 **	0.67 **

Note. PS = pollution sensitivity; PCI = pollution–causes–illness; PA = pollution acceptance; CEA = community environmental action; PEA = personal environmental action; K-EHL = The Korean version of the Environmental Health Literacy scale; K-EHEP= Korean version of the Environmental Health Engagement Profile; ** *p* < 0.01.

## Data Availability

Not applicable.
